# The Fallacy of Year-Round Breeding in Polyphagous Tropical Fruit Flies (Diptera: Tephritidae): Evidence for a Seasonal Reproductive Arrestment in *Bactrocera* Species

**DOI:** 10.3390/insects13100882

**Published:** 2022-09-28

**Authors:** Anthony R. Clarke, Peter Leach, Penelope F. Measham

**Affiliations:** 1 School of Biology and Environmental Science, Queensland University of Technology (QUT), P.O. Box 2434, Brisbane, QLD 4001, Australia; 2 Horticulture and Forestry Science, Department of Agriculture and Fisheries, P.O. Box 652, Cairns, QLD 4870, Australia; 3 Horticulture and Forestry Science, Department of Agriculture and Fisheries, Ecosciences Precinct Dutton Park, P.O. Box 267, Dutton Park, QLD 4102, Australia

**Keywords:** *Bactrocera tryoni*, *Bactrocera dorsalis*, reproductive arrest, biological invasion, population phenology, cold tolerance, monsoon rainforest

## Abstract

**Simple Summary:**

*Bactrocera* fruit flies are major pests of horticulture in tropical parts of the world and are highly invasive. Able to breed in many different fruit types, and living in hot to warm climates where temperature is not limiting, it is assumed that these flies breed continuously in their native environment. However, *Bactrocera* are native to monsoonal rainforests, where the mature fruit needed for breeding is largely absent for four to five months a year during the dry season. Reviewing literature and published population graphs of these flies, we argue that there is evidence to suggest that these flies undergo a reproductive arrest during the dry season when breeding hosts are scarce. We believe females stop or limit reproduction through a diapause or quiescence mechanism, so extending their life-span during the unfavourable breeding period. Once through that period they then switch their life-history strategy to focus on reproduction. Evidence is that this behaviour continues in invaded and agricultural systems and is not just restricted to rainforests. We cannot confirm this hypothesis with the information available, but because of its potential significance in managing these pests we urge that targeted research be carried out to confirm or deny the hypothesis.

**Abstract:**

The genus *Bactrocera* (Diptera: Tephritidae) is endemic to the monsoonal rainforests of South-east Asia and the western Pacific where the larvae breed in ripe, fleshy fruits. While most *Bactrocera* remain rainforest restricted, species such as *Bactrocera dorsalis*, *Bactrocera zonata* and *Bactrocera tryoni* are internationally significant pests of horticulture, being both highly invasive and highly polyphagous. Almost universally in the literature it is assumed that *Bactrocera* breed continuously if temperature and hosts are not limiting. However, despite that, these flies show distinct seasonality. If discussed, seasonality is generally attributed to the fruiting of a particular breeding host (almost invariably mango or guava), but the question appears not to have been asked why flies do not breed at other times of the year despite other hosts being available. Focusing initially on *B. tryoni*, for which more literature is available, we demonstrate that the seasonality exhibited by that species is closely correlated with the seasons of its endemic rainforest environment as recognised by traditional Aboriginal owners. Evidence suggests the presence of a seasonal reproductive arrest which helps the fly survive the first two-thirds of the dry season, when ripe fruits are scarce, followed by a rapid increase in breeding at the end of the dry season as humidity and the availability of ripe fruit increases. This seasonal phenology continues to be expressed in human-modified landscapes and, while suppressed, it also partially expresses in long-term cultures. We subsequently demonstrate that *B. dorsalis*, across both its endemic and invasive ranges, shows a very similar seasonality although reversed in the northern hemisphere. While high variability in the timing of *B. dorsalis* population peaks is exhibited across sites, a four-month period when flies are rare in traps (Dec–Mar) is highly consistent, as is the fact that nearly all sites only have one, generally very sharp, population peak per year. While literature to support or deny a reproductive arrest in *B. dorsalis* is not available, available data is clear that continuous breeding does not occur in this species and that there are seasonal differences in reproductive investment. Throughout the paper we reinforce the point that our argument for a complex reproductive physiology in *Bactrocera* is based on inductive reasoning and requires specific, hypothesis-testing experiments to confirm or deny, but we do believe there is ample evidence to prioritise such research. If it is found that species in the genus undergo a true reproductive diapause then there are very significant implications for within-field management, market access, and biosecurity risk planning which are discussed. Arguably the most important of these is that insects in diapause have greater stress resistance and cold tolerance, which could explain how tropical *Bactrocera* species have managed to successfully invade cool temperate regions.

## 1. Introduction

Endemic to the tropical rainforests of South-east Asia and the western Pacific [1], *Bactrocera* Macquart species are invasive in both tropical and temperate zones [2,3] where they are highly disruptive to crop production and trade [4]. While not yet established in either mainland Europe or North America, *Bactrocera dorsalis* (Hendel) (Oriental fruit fly) is regularly detected as incursive populations on both continents [5,6,7] and has the potential to establish in both under current [8] and future climates [9]. In Australia and China, which are large enough to have distinct tropical and temperate regions, *Bactrocera tryoni* (Froggatt) (Queensland fruit fly) and *B. dorsalis*, respectively, are endemic to their tropics but invasive in temperate areas [10,11]. 

Because of their impact and invasive potential, a large amount of pest management and biosecurity risk reduction activities are carried out against *Bactrocera* species. Examples of the types of biosecurity activities taken against *Bactrocera* include predictive range mapping [8,12,13], optimisation of trapping arrays [14,15], post-entry spread models [16], landscape mapping [17], predictive crop impacts [18], and infield management [19,20]. Other than climate-matching models which make no assumptions about underlying organism biology, nearly all such studies make, either explicitly or implicitly, a key critical assumption: that the polyphagous *Bactrocera* species breed continuously so long as environmental factors, most commonly temperature and breeding hosts, are not limiting. An explicit statement to this effect is made by Baker et al. [5]: “*The ‘dorsalis complex’ is one of the most destructive pest species complexes in global fruit production due to polyphagy, invasiveness, high reproductive potential, multivoltinism and continuous activity throughout the year*” [our emphasis]. Implicitly it can be seen in models where temperature is the primary driver of population cycles [21,22,23,24], or in population models which seek correlation between fly numbers and environmental variables [25,26,27]. 

Year-round breeding in tropical insects assumes that reproduction is limited by neither temperature nor host availability, yet despite ambient temperatures in the tropics being high and relatively stable, tropical insect populations can show strongly seasonal dynamics [28,29,30]. Many tropical forest communities are ecologically driven by monsoonal wet and dry seasons where predictable changes in rainfall and relative humidity drive the timing of plant growth, flowering and fruiting [31,32] which, in turn, drive the population dynamics of rainforest insects [33,34] and higher animals [35]. Further, and in parallel with temperate insects [36,37,38], tropical insects in monsoonal regions often have evolved diapause and quiescence mechanisms which allow them to cope with the long dry season when oviposition and feeding substrates (e.g., new shoots, young foliage, flowers, fruit) are not available [39,40,41,42]. Tropical diapause is not just restricted to the first trophic level but extends to higher-tropic level insects such as parasitoids [43]. So common is tropical diapause that for both of the dipteran families Drosophilidae and Sacrophagidae it is possible that their well-studied diapause mechanisms first evolved in the tropics [44,45], while radiations of tropical butterflies have also been linked to their ability to diapause [46].

In this forum paper, focusing initially on the Queensland fruit fly but then expanding to Oriental fruit fly, we identify ‘clues’ which suggest that, like many other tropical insects, these flies may also have a diapause or quiescence mechanism that leads to a seasonal reproductive arrest, plus other adaptative mechanisms which, while initially evolved for surviving and maximising reproduction in their endemic rainforest habitat, are still expressed in human-modified landscapes. The available data is insufficient to confirm that such adaptations exist, but we consider the circumstantial evidence is sufficient to justify targeted research to confirm or deny the hypothesis. If confirmed, the findings would greatly impact on population modelling and risk analysis for these species, a topic which we address in the last section of the paper.

### 1.1. Phenology of Queensland Fruit Fly

#### 1.1.1. Background I: Queensland Fruit Fly and Its Temperate ‘Overwintering’

*Bactrocera tryoni*, the Queensland fruit fly or just Qfly, is historically native to the tropical and subtropical east-coast Australian rainforests [47] but is now a horticultural pest in both tropical and temperate parts of Australia [10,48] and is invasive in Oceania [19]. The movement of the species into temperate Australia occurred gradually during the 20th century and, up to the 1970s, much of the phenology work on the species focused on the question of how a tropical fly survived cold temperate winters. The lack of a pupal diapause but adult survival over winter has been confirmed many times [49], with the physiological basis of the adult overwintering researched intensively during the 1960s and 1970s by Drs Alfred (Alfie) Meats and Brian Fletcher. Fletcher identified that adult Qfly generated in the late autumn/early winter did not sexually mature until the following spring, while females that were already mature and mated leading into winter resorbed their ovaries and emptied their spermatheca during winter [50,51,52]. The resorption of ovaries was a presumed adaptive response for energy saving and reallocation during cold periods when food foraging was restricted [50]. Meats focused more broadly on how a tropical insect could survive in temperate areas and carried out pioneering research on insect cold acclimation [53,54,55,56,57,58]. As part of his research he developed an early bioclimatic model for Qfly, predicting that the species could have between seven to nine generations per year in subtropical and tropical Australia as temperature was rarely limiting [21]. Meats and Fletcher invariably interpreted their results in terms of temperate biology; for example Meats [56,59] and Meats and Fitt [60] discuss Qfly seasonal cold acclimatization in terms of frost tolerance, without asking why a tropical insect should have an evolved mechanism for avoiding frost.

Despite the extensive research carried out on Qfly overwintering, the species’ phenology in temperate Australia can still not be fully explained. Using temperature to explain Qfly ovarian development, Fletcher [50] acknowledged that while a day-degree model based on a critical threshold temperature of 13.5 °C could adequately explain ovarian resorption or maturation for most of the winter period, there were several datasets where it failed to predict ovarian changes leading into, or coming out of winter. A cohort-based Dymex model for *B. tryoni*, incorporating all phenological and demographic knowledge then available for Qfly, had similar problems with the winter period: “*The five simulations all point to the need to understand what causes fruit flies to over-winter… We explored numerous mechanisms, including accumulating degree-days below various thresholds, accumulating insufficient warmth (i.e., degree-days of above various thresholds), and accumulating net cold (i.e., number of degree-days below a threshold temperature minus the number of degree-days above the same temperature threshold), but could find no mechanism that would work consistently… The various mechanisms tested indicate that over-wintering is not just temperature-dependent, but is probably linked to a variety of factors, including temperature, fruit availability and fruit suitability. … Until the mechanisms determining the onset and the ending of over-wintering are understood, it will be difficult to improve on the model predictions of fly numbers from about May to August”* [22]. The model of Yonow et al. [22], developed and validated against temperate Qfly phenology data, was subsequently shown to fail when run against Qfly tropical phenology data [61].

#### 1.1.2. Background II: Queensland Fruit Fly and Its Tropical Phenology 

**Figure 1:**
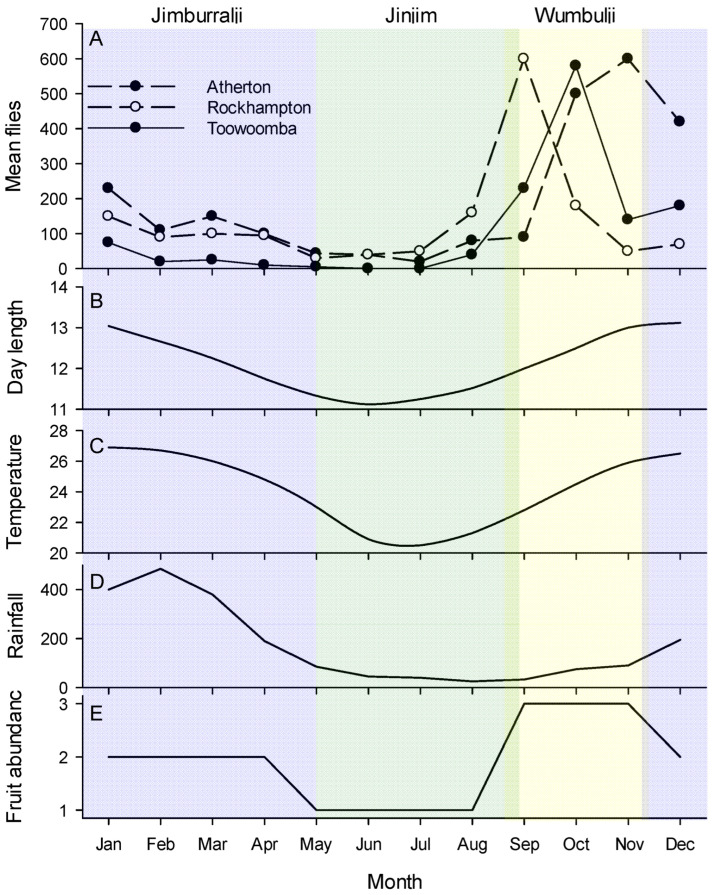
(**A**) Illustrative phenology patterns of *Bactrocera tryoni* in Australia against (**B**–**E**) climate variables and seasonal patterns. Atherton is in the wet tropics of Far-north Queensland (graph is the mean of four years data, 1955–1958); Rockhampton is in a dry zone located on the Tropic of Capricorn (mean of two year’s data 1955–1956); Toowoomba is a subtropical site in southern Queensland (mean of four year’s data 1950–1953). Rainfall (mm), temperature (°C) and day length (hrs) are for Cairns, a tropical city very near Atherton. Fruit abundance is a simple relative estimation (low, medium, high) of the amount of mature fruit in the tropical Australian rainforest forest based on literature [66]. The colours represent the local seasons of the Cairns district as recognised by the Yirrganydji people of Far-North Queensland. From left to right these are: (blue) Jimburralji [cyclone time], (green) Jinjim [cool time], (yellow) Wumbulji [hot and humid time]. Phenology data from Muthuthantri et al. [61], weather data from Australian Bureau of Meterology (www.bom.gov.au accessed on 7 September 2022), indigenous calander from http://www.bom.gov.au/iwk/calendars/yirrganydji.shtml (accessed on 7 September 2022).

In the Australian tropics and subtropics, in historical datasets [61], modern production systems [62,63] and native rainforest systems [64], Qfly shows a very significant depression in trap catch in the four-month period from May to August. From late August and early September populations begin to increase dramatically until they peak by November, at which time they begin a gradual decline, sometimes with a second peak, towards May (Figure 1). In the southern hemisphere May through August are late autumn/winter months and so the seasonal disappearance of Qfly is not, perhaps, unexpected. However, in tropical Queensland ‘winter’ is cool only relative to the hot months of the remainder of the year and, as identified by both Meats [21] and Yonow and Sutherst [65], temperature should not be limiting to *B. tryoni* population growth in the tropics and subtropics even in the winter months, let alone during the summer and early autumn when populations are nevertheless declining. 

### 1.2. So If Its Not Temperature, What Is It? Evidence for Reproductive Diapause in B. tryoni

While relying heavily on inference we believe there is sufficient literature evidence to suggest that *B. tryoni* has a complex reproductive physiology, involving seasonal life-history trade-offs between longevity and reproduction, which explains its phenology and demography in both tropical and temperate areas. We consider the phenology to be adaptive for its endemic, tropical monsoonal rainforest habitat, but that it continues to be expressed in human-modified landscapes and even shows some expression after long-term laboratory rearing. In the following sections we first explain the endemic habitat of the species and demonstrate that the phenology of the fly closely aligns with the local seasons as recognised by first nations owners. We then present demographic data which shows that the fly has longevity and reproduction patterns which can (at least partially) act independently of temperature and explain how the fly is capable for living for periods between fruiting seasons and then maximally utilising hosts when they are available. 

#### 1.2.1. Native Habitat Conditions

*Bactrocera tryoni*, as for most other members of the genus, is considered endemic to tropical rainforest [47]. The Australian east-coast rainforests are monsoonal, with the wet season running from early December running through to March [67]. As recognised by the Yirrganydji people of Far-North Queensland, the seasonality of the tropical wet forests is complex consisting of not just Kurrabana (wet season, December to May) and Kurraminya (dry season, May to December), but also within those Jinjim (cool time, May to late August), Wumbulji (hot and humid time, September to November) and Jimburralji (cyclone time, December to May) (Figure 1 and http://www.bom.gov.au/iwk/calendars/yirrganydji.shtml). These rainforests have peak flowering during Wumbulji [32], while insects are most abundant from November through to February [34]. Fruit development rate is variable and at least some fruit is available year-round [68], but in the tropical wet forest peak fruit fall is between August and February [66]. In the subtropical east-coast forests the fruiting cycle is a little later and ripe fruit is scarce from June to October [69]. Important to all aspects of the ecology of tropical tephritids [70,71,72], fruit and leaf phylloplane microbial communities are also seasonal and based on work in other tropical systems would be expected to grow during the hot and humid time of Wumbulji [73,74,75]. The temporal alignment of the Yirrganydji calendar with the phenology of *B. tryoni* in Atherton (Figure 1), which sits just outside Yirraganydji country, is extraordinary. The period of population increase occurs during the hot and humid period of Wumbulji, the period of population decline occurs during Jawarranyji and Jimburralji, and the period when the population is effectively absent from traps is the cool time of Jinjim. 

#### 1.2.2. Demography of the Queensland Fruit Fly

Queensland fruit fly are known to be long lived. In the field in cool temperate Australia, 50% of adult flies emerging from mid-April to mid-May were still alive 100 days later, and 10% still alive 160 days later [76]. However, importantly, adult longevity is not just dependent on ambient conditions but can vary with time of year even when temperature is constant. Under constant laboratory conditions, Tasnin et al. [77] demonstrated that *B. tryoni* adults collected as maggots from field infested fruit and then held under the same constant laboratory conditions had different longevities: flies collected in mid-August lived up to 240 days, flies collected in mid-May for 200 days, and flies collected in mid-September and mid-March less than 120 days. This seasonal difference in longevity even occurs in long-term culture lines. In an analysis of 16 years of quality assurance data from a constant-condition Qfly mass-rearing facility, adult lifespan and adult longevity under nutritional stress (no food or water) were both significantly affected by month, being longest in June, July and August, and shortest in December and January [78]. Dominiak et al. [79] provides a second stress-survival data set for adult Qfly with very similar seasonal results. Dominiak et al. [80] links increased adult longevity during winter with seasonal declines in Qfly pupal weight (in cultured flies) and adult weight (in both wild flies and culture flies) and suggests the weight/longevity relationship is a mechanism for surviving winter. However, like the earlier work of Meats and Fletcher, the analysis done by Dominiak et al. [80] is done within a temperate, four-season year and does not consider alternative interpretations of the data—for example weight loss during a dormancy period.

As for adult longevity, there is also evidence that juvenile development, sexual maturation and egg development in *B. tryoni* are not fully explained by temperature and may be seasonally influenced. Working in the environs of Sydney, in cool temperate Australia, Pritchard [81], Fletcher [50] and Meats and Koo [58] all studied *B. tryoni* sexual maturation and ovarian development with respect to temperature, with Fletcher developing a day-degree model with a critical threshold temperature of 13.5 °C to explain ovarian development (above 13.5 °C) or egg resorption (below 13.5 °C). However, these studies also identified issues that temperature could not explain. Both Pritchard and Fletcher reported flies not sexually maturing in the autumn when temperatures should not have been limiting for maturation; while Pritchard reported rapid sexual maturation of flies in August, a month for which Fletcher’s model predicted minimal ovarian development. In the same region but looking at different questions, Bateman and Sonleitner [82] could also not explain why *B. tryoni* populations greatly declined in mid-March despite temperature and hosts not being limiting. At a subtropical coastal site where temperature should not be limiting for Qfly development [21] and hosts were always available [83], Tasnin et al. [77] found demographic data that could not be explained by temperature, detecting an autumn through winter breeding cessation which meant that instead of the site supporting the seven generations per year predicted by both Meats [21] and Yonow and Sutherst [65] based on day degree accumulation, it had only three. Tasnin et al. [77] also reported that the population surviving at the end of winter consisted primarily of old individuals, which poses a paradox. While old to very old *B. tryoni* can lay viable eggs, their daily egg production is very limited [84]. How these individuals, if they dominate in the population, can generate an explosive increase in the population within a matter of weeks is not clear. 

#### 1.2.3. So What Do We Think Is Happening?

We suspect an understanding of the phenology of *B. tryoni* has been confounded by the very early [85,86] and ongoing [22,50,56,80,82] pattern of explaining Qfly’s phenology within the context of a temperate, four-season year, rather than within the context of its evolutionary endemic environment—the monsoonal tropical rainforest. Once a pupal diapause in *B. tryoni* was dismissed and adults had been shown to be able to survive in the field all year round [49] no attention was paid to other forms of arrested development, yet we consider that an adult reproductive quiescence or reproductive diapause (sensu [87]) best explains the data. 

An arrest in reproduction, through diapause or quiescence, is well documented in tropical insects and involves an arrest in oocyte development and an absence of oviposition [40]; for species with a female reproductive diapause, a corresponding male diapause may or may not occur [88]. Additional to the direct reproductive effect, reproductive arrest can also result in significantly delayed senescence [89], increased stress resistance and enhanced somatic maintenance [90], and decreased metabolic rates [38]. While reproduction is halted, the adult insect may retain mobility and feeding [91,92] or they may aggregate and have limited feeding [39,41]. Reproductive diapause may be facultative rather than absolute: in a study of the tropical butterfly *Hypolimnas bolina* L., Pieloor and Seymour [93] found 18% of females did not enter diapause which they argued was an adaptation to take advantage of the unpredictability of tropical seasons. For tropical environments the cues associated with the entry and exit of the different eco-physiological phases of diapause (sensu [37]) are complex and can include both increasing and decreasing photoperiod, rainfall, temperature, humidity and the availability of hosts [40].

It is probable that an inadequate knowledge of *B. tryoni*’s tropical phenology, and of tropical diapause/quiescence, led earlier workers to misinterpret their findings on ‘winter’ ovarian change in the species. Quoting from the Introduction of Fletcher [51]: “*In the northern parts of its range* B. tryoni *breeds more or less continuously throughout the year, but in the southern part breeding is curtailed by low temperatures during the winter months… …They do not have a true diapause, but they become sexually inactive and in the females developing oocytes are resorbed*”. In the 1970s no published tropical phenologies of *B. tryoni* were available for Fletcher to compare with, and even by the mid-1980s diapause was still “*frequently viewed as a developmental strategy unique to insects of the temperate zone*” [40]. It is thus not surprising that the reproductive shut-down observed by Fletcher [50] and Meats and Khoo [58] was interpreted by them as a direct cold temperature effect, rather than a tropical dry-season reproductive arrest which temporally over-laps with the temperate winter. However, an interpretation of reproductive arrest better explains the reported early shutdown in *B. tryoni* breeding and/or ovarian development prior to the onset of cold winter [50,81,82], ovarian development commencing again before the winter cold is over [81], and the breeding cessation/suppression now known to occur in the tropics [61,62,63,64,77]. As an example of a similar insect frugivore system where modern knowledge has allowed interpretation not possible in the 1970s, reproductive arrest in *Drosophila melanogaster* is now considered a trait which evolved in the tropics rather, than as originally assumed, in the temperate zone [45]. 

Further to explaining the direct reproduction effects, a reproductive arrest helps explain other anomalies in the demography and physiology of *B. tryoni*. Insects in reproductive arrest age more slowly than when not in arrestment [89], which would explain the seasonal differences in adult longevity under constant temperature conditions [77,78]. Tropical insects in reproductive arrest also have to suppress metabolism as they cannot rely on cold temperatures to minimise the consumption of their internal energy reserves [38], which would explain *B. tryoni*’s greater longevity under nutritional stress during the cooler months [78,79]. Meats [59] also records that metabolism rates of ‘cold acclimated’ *B. tryoni* were reduced compared to non-acclimated flies, a result opposite to which he was expecting to see, but unfortunately his cited sources are unpublished student theses which are no longer available.

Additional to aiding longevity, we also believe a reproductive arrest helps explain the sudden build-up of *B. tryoni* populations during the short, hot-humid period of Wumbulji. Yap et al. [94] identified a highly unusual reverse-aging mechanism in *B. tryoni*. They found that adult female Qfly of up to 30 days of age (the longest of their test periods) which had been denied yeast, sugar and access to mates, had reversed actuarial aging (i.e., they became physiologically young again) if supplied diet that allowed sexual maturation and were then allowed to mate. Females treated in this way subsequently had longevity and egg-production equivalent to newly matured flies. The authors considered the presence of male accessory gland fluid as the likely trigger for the effect which they proposed “…*allows Qflies to synchronize reproduction and mortality schedules*.” In the context of a putative reproductive dormancy in *B. tryoni*, Fletcher [50] identified that female *B. tryoni* empty their spermatheca at the same time as resorbing their ovaries, but could not provide a reason why. However, if those females survive the shut-down period and become active again in August, then new mating and filling of the spermatheca should act to make them physiologically young again, maximising their potential to utilise seasonally available fruit resources and so start the dramatic population increases seen in trap data.

In summary, we feel there is sufficient evidence to support a case that *B. tryoni* has the capacity to enter a reproductive arrest, which in temperate areas allows it to survive cold winters but is almost certainly an evolved response for surviving the tropical late dry season when breeding hosts are scarce. In comparison to the well-studied reproductive arrest in *Drosophila melanogaster* Meigen, the phenotypic expression of the putative reproductive arrest in *B. tryoni* is almost identical (Table 1). 

#### 1.2.4. What Don’t We Know?

A lot. As for Rossi-Stacconi et al.’s [92] paper proposing a reproductive diapause in *Drosophila suzukii* in northern Italy, we consider that there are sufficient lines of evidence to suggest a reproductive arrest in *B. tryoni*. However, we reiterate that the interpretation is based on inductive reasoning and specific experiments need to be run to confirm or deny the hypothesis. *If* a reproductive arrest is confirmed, then future research needs to: determine if it is a diapause or a quiescence; explore the triggers for arrest induction, initiation and termination; if there is a diapause component then if a post-arrest quiescence stage occurs; and if arrest is obligatory or facultative (terminology follows [37]). Observation suggests that a *B. tryoni* reproductive arrest would be facultative rather than obligatory, as eggs can be recovered from the field all year round even if at reduced levels [83], while laboratory cultures can be continued all year round even if culture quality-assurance attributes vary seasonally [78]. The ability to culture flies year-round suggests an exogenous environmental cue triggering reproductive arrest that is not experienced by lab cultures, or that a percentage of a Qfly population never enters reproductive arrest and these individuals become selected for within culture lines: both signs of facultative diapause [95]. Variation in the initiation of timing of *B. tryoni* population peaks around Australia [22,61] is suggestive that if there is a true reproductive diapause then there may be a post-diapause quiescent stage where flies are a primed for sexual activity but wait for an exogenous cue before becoming fully active [37,40], or alternatively that the entire reproductive arrest is a quiesence driven entirely by exogenous cues. Additionally, the direct effects of local temperature will influence populations regardless of whether individuals are in reproductive arrest or not and so also needs to be considered. 

### 1.3. Is There a Reproductive Arrest in Other Dacine Fruit Fly Species?

Fitt [96,97] presents strong evidence that *B. opiliae* (Drew & Hardy), a monophagous non-pest fruit fly of the dry tropics of northern Australia, survives the months between the annual fruiting cycle of its host as a quiescent adult and that the fly’s maturation rate is greatly reduced during the quiescent period. Fletcher [98], in his seminal review of the Dacinae, states that “*No known dacine have a true diapausing stage, but the adults of some species are able to pass unfavourable periods of the year in a facultative reproductive “diapause” during which they aggregate in suitable refuges and remain in or revert to a sexually immature state*.” Thus Fletcher recognised 35 years ago that at least some dacines have a reproductive arrest, even if at the time it did not count as a ‘true’ diapause. However, of the three references that Fletcher cites in support of his statement only Fitt [96] provides direct evidence for reproductive arrest: the other two papers provide evidence for cool/dry season aggregations of adult *B. zonata* (Saunders) [99], and multiple African *Dacus* species [100] but they make no comment about the flies’ reproductive state. So what evidence is there that other *Bactrocera* species, especially polyphagous species such as *B. dorsalis*, might have a *B. tryoni* like reproductive arrest, versus having “*continuous activity throughout the year*” [5].

**Figure 2:**
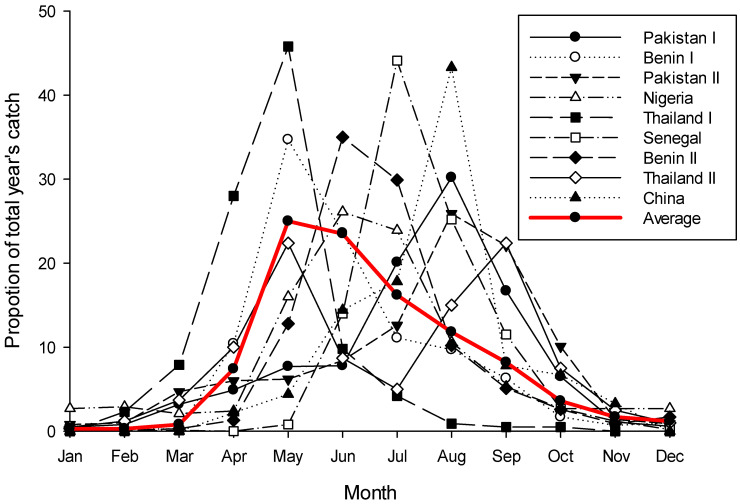
Yearly phenology curves for *Bactrocera dorsalis* for nine sites across the northern hemisphere, plus the average plot of those nine. Data for plots was read from tables or graphs in the source papers and then transformed as a proportion of the total year’s catch for that site. Variance data from the original sources is not included, and where data had to be read from graphs exact values are not guaranteed. Sources [106,107,108,109,110,111,112,113]; these sources were selected simply for the ease by which their data could be read.

While very noisy, phenology data for *B. dorsalis* from across its native and invasive range in the northern hemisphere clearly shows that the fly does not have continuous activity throughout the year; rather it shows a phenology pattern very similar to *B. tryoni*—except reversed (Figure 2). Following a period of very low or zero catches running from December through to March the population rapidly increases over the next two to three months, before gradually declining back to the December low. There is a great deal of variation in this pattern across sites, but three key elements are consistent: (i) most sites have only one peak per year, very rarely two; (ii) there is a consistent four-to-five month period when flies are rare or absent from traps; and (iii) population peaks for individual sites during the active period are not normalised around the warmest months of the year which would be expected if temperature was the dominant system driver. In the tropics a strong population seasonality, with an absence or near absence of adults at a regular time of the year, is considered good evidence for a diapause [29,101]. Further, it should be noted that when sampling sites are on, or very near the equator there is no apparent seasonality in *B. dorsalis*’s phenology (see Figure 6c,g in De Villiers et al. [8], both sites are within 1.5° of the equator), while south of the equator the phenological pattern is reversed with the population peaking around January/February (see Figure 6b in De Villiers et al. [8] and Theron et al. [102]). If there is a diapause in *B. dorsalis*, and if it is influenced by a critical day-length, then a reversed pattern either side of the equator would be expected to be seen, as would no pattern on the equator as insects are considered unable to measure the subtle day-length changes which occur ±5 degrees of the equator [103,104,105].

In Hawaii, in addition to detecting seasonality in adult abundance, Bess and Haramoto [114] also identified that fruit infestation by *B. dorsalis* was seasonal, with maximum infestation between April and September, and minimum between November and March. This was despite fruit being available for breeding all year round. Subsequently, Haramoto and Bess [115] showed that ‘summer’ infestation of guava by *B. dorsalis* was up to 80 times more than ‘winter’ infestation of guava. Further, Newell and Haramoto [116] documented that *B. dorsalis* adult peaks and oviposition peaks did not align. Rather, the rarer adults early in the season were laying as many as 30 times more eggs than the more abundant adults later in the season. “*Maximum egg production occurs at times of increasing or maximum fruit abundance and the fly populations present at such times are usually of only moderate size. They appear to comprise flies which for the most part have survived in the field for considerable periods of time and which become concentrated in areas in which there is a maturing crop of fruit. Declining fruit crops are paralleled by declining ovipositional activity*.” While the ‘cool’ winter months have been used to explain the seasonality of *B. dorsalis* fruit infestation in Hawaii from as early as 1961 [114], winter versus summer temperatures vary minimally on the Hawaiian Islands (winter average daily min-max 18.3–26.1 °C; summer 21.1–28.9 °C, https://www.weather-us.com/en/hawaii-usa-climate) and all are within the favourable to optimal range for *B. dorsalis* development, survival and fecundity [24,117,118]. Thus ‘winter’ cannot explain the seasonality of oviposition by *B. dorsalis* in Hawaii. Rather, the phenology and demography of *B. dorsalis* in Hawaii appears to align with our model for *B. tryoni*, that in the field flies seasonally switch their physiological investment between longevity and reproduction and this has continued to occur even in human-modified landscapes where hosts are now always available [119].

Over the last decade numerous researchers have applied increasingly sophisticated multivariate, neural network and fuzzy-logic analyses to seek correlations between *B. dorsalis* population numbers and weather and/or crop variables [25,26,27,120,121,122,123,124,125]. In some cases correlations have been very high and it could be argued that such analyses are evidence against *B. dorsalis* having anything more than a ‘simple’ weather/host-availability driven phenological cycle. While this may be so, looking closely at the papers we do not think this is the case. Firstly, some of these papers model changes of fly abundance within a single cropping season [26], or even as little as one week ahead [123], where we would expect daily weather variation to play an important part in explaining trap-catch variation. Secondly, important correlative variables are not always those that might be expected in a ‘weather and breeding-up-within-crop’ phenology. Notably, Kamala Jayanthi and Verghese [120], Kamal Jayanthi et al. [122] and Ibrahim et al. [25] all demonstrate strong correlations between fly trap catches and the abundance of immature fruit, but not the abundance of mature fruit. Thus these studies show that fly populations are correlated with the crop still to come, rather than being directly correlated with breeding within that crop, a subtle but important difference that reinforces the early work of Newell and Haramoto [116] in understanding the difference between adult abundance and adult reproduction. Finally, as some of these models use ‘hidden layer’ components and are only tested against internal data [26,27,121], evaluating their predictive capacity against independent data sets is not possible.

Summarising this section, we feel that the many published phenologies of *B. dorsalis* demonstrating strong seasonality (Figure 4), evidence of differential reproductive investment at different times of the year [116], and populations that are best correlated with the developing crop rather than the mature crop [120] are all clues that this species may also have a seasonally complex phenology akin to what we think is happening in *B. tryoni*. Certainly for *B. dorsalis* there is not continuous activity throughout the year in the tropics and that belief should be dismissed. What about other dacine species? Unfortunately for most species, even other major pest species, there is simply insufficient knowledge to know.

## 2. Why Is All This Important?

Additional to simply better understanding the biology of dacine fruit flies, confirming or denying the presence of repeatable, seasonal changes in the reproductive activity of pest fruit flies, happening independently of temperature or host availability, has significant ramifications for many areas of plant biosecurity. These are addressed below under the sections: in-field control, market access, and risk reduction.

*In-field control*: Confirming if flies are investing more or less in reproduction versus longevity at different times of the year, especially if there are critical cues that cause switching between the two, would enhance the ability to create predictive population models for pest management. We are aware of only three predictive population models for polyphagous *Bactrocera* species that have been tested against independent data sets: one for *B. tryoni* [22] and two for *B. dorsalis* [124,126]. All three models consistently over-predicted the size of fly populations for large periods of the year. If seasonal changes in longevity/reproduction trade-offs are occurring, then this over-prediction of populations will not just be because of the reproduction predicted to occur but not occurring during a reproductive shut down period, but also because investment in egg production appears not to be consistent even across the breeding period and appears to decline after only a few months. Even without better predictive modelling, benefits for in-field control are still gained if a reproductive arrest is confirmed. If populations do reset themselves demographically each year, as suggested by Yap et al. [94] and demonstrated in the field by Tasnin et al. [77], then it becomes far easier to time the application of behaviour-based controls, such as protein bait sprays or SIT, so that they target populations when most demographically susceptible. For example, *B. tryoni* in the field have minimal protein hunger late in season and respond very poorly to protein bait sprays [127], but with the demographic knowledge gained by Tasnin et al. [77] we might now reasonably predict that flies will be very protein hungry in September/October as they rebuild nutritional reserves and invest heavily in egg production.

*Market access*: Crops which are harvested during a period of reproductive shutdown may need reduced levels of pre- and post-harvest risk reduction to access markets. For example, ‘winter’ strawberries exported from Queensland to other Australian states prior to the 10th of August have a reduced in-field risk reduction step compared to those exported after the 10th [128]: this is based on the very low risk of fruit fly infestation prior to mid-August, but an increasing risk of fruit fly infestation by late August [129]. Similarly, market access of tomatoes and capsicums from the tropical Queensland horticultural production area of Bowen has been argued (although not gained) based on a ‘winter’ window running from March to early August when flies are largely absent from traps and fruit infestation is almost entirely absent [63]. Reinstatement dates, i.e., the time after the last detection of a regulated pest before an area is declared ‘pest free’, may also be impacted if populations have a cessation in breeding which is independent of temperature. For market access purposes, much more detailed information would be needed about the role (if any) of predictable environmental cues in a reproductive arrest, so regulatory dates could be set with confidence and transparency.

*Risk reduction*: If polyphagous *Bactrocera* are confirmed to have a reproductive arrest then there are several issues to consider with respect to biosecurity risk reduction. Many planning activities rely on underlying population models [15,16,130,131] and the accuracy of the outputs of those models will impact on the robustness of preparedness planning and response. However, beyond modelling which has already been discussed, the proven presence of a reproductive arrest is of particular importance for risk assessments based on climatic suitability [5,132,133,134]. The presence of a reproductive arrest which allows adults to survive a stressful period without hosts will also provide the flies greater ability to survive stressful periods when the stress is caused by something different—notably cold. The work of Meats [54,55,56,57,135], while confounded by his assessment of the drivers of what he observed, clearly shows that *B. tryoni* has high cold tolerance during its period of reproductive quiescence. This is most likely due to the fact that insects in diapause, even tropical diapause, have greater cold tolerance [136] and stress handling capacity [38,40]. The migration of *B. dorsalis* into cold temperate China [11], not originally predicted by climate driven models [137], may be partially explained by this mechanism. The rapid global invasion of *Drosophila suzukii* and its ability to tolerate harsh winter conditions has been attributed to its facultative adult diapause [92], and this attribute may unfortunately prove to be just as important for the spread of invasive *Bactrocera*.

## 3. Conclusions

We are not the first to suggest that dacines may have an adult reproductive arrest. Direct statements to that effect are already in the literature [51,96,97,98], while indirect evidence is hidden in plain sight in multiple publications spanning the last 60 years. Hernandez-Ortiz and Perez-Alonso [138] also suggest an adult diapause to explain the phenology of *Anastrepha* species in tropical rain forests of Mexico, while Aluja et al. [139] touch on the potential of “*aestivating or long-lived adults*” to explain seasonality in Mexican *Anastrepha obliqua* (Macquart). Species of the genus *Rhagoletotrypeta* survive the long periods between host availability on the tropics and subtropics through a pupal diapause which can extend from eight to 12 months [140]. Further, the now well-recognised diapause known to occur in many fruit fly parasitoid species [141,142,143] is also suggestive that their hosts must have a regular breeding break, as parasitoid life histories and seasonality are closely linked to those of their hosts [144,145]. However, the current international fruit fly and plant-biosecurity communities, including ourselves until very recently, appear to have missed these clues entirely. Because no researcher or research group has ever explicitly tested for a reproductive arrestment in a *Bactrocera* species (although the work of Fitt comes close) we fully accept that this remains a hypothesis waiting to be tested, not a proven fact. The purpose of this paper is not to convince people that a reproductive arrest exists, but to raise the experimental confirmation or denial of a reproductive arrest in *Bactrocera* as a priority research area which has significant flow-on implications for both in-field management and invasion preparedness.

## Figures and Tables

**Table 1:** Comparison of the recognised non-dormancy and dormancy reproductive states of *Drosophila melanogaster* with the proposed non-dormancy and dormancy reproductive states for *Bactrocera tryoni*. The information for *D. melanogaster* is extracted from Figure 3 in Flatt et al. [87] and is fully referenced by them: the references provided are just for *B. tryoni*.

*Drosophila melanogaster*		*Bactrocera tryoni*		
Normal Reproductive Function	Reproductive Dormancy	Normal Reproductive Function	Reproductive Dormancy	References
Normal mature ovary	Non-vitellogenic, immature ovary	Normal mature ovary	Non-vitellogenic, immature ovary	[50,81]
Normal fecundity	Ovarian arrest	Normal fecundity	Ovarian arrest	[58]
Relatively low stress resistance	Stress-resistant	Relatively low stress resistance	Stress-resistant	[78,79]
Normal metabolism	Reduced metabolism	Normal metabolism	Reduced metabolism	[59]
Quite short lifespan	Long lifespan	Shorter lifespan	Long lifespan	[77,79]
	Able to overwinter		Able to overwinter	[51,76]

## Data Availability

No new data was generated for this paper. Data presented in the figures is available from the sources cited within the figure legends.

## References

[1] Drew R.A.I. (2004). Biogeography and speciation in the Dacini (Diptera: Tephritidae: Dacinae). Bishop Mus. Bull. Entomol..

[2] Papadopoulos N., Shelly T., Epsky N., Jang E.B., Reyes-Flores J., Vargas R. (2014). Fruit fly invasion: Historical, biological, economic aspects and management. Trapping and the Detection, Control, and Regulation of Tephritid Fruit Flies.

[3] Ekesi S., Mohamed S.A., De Meyer M. (2016). Fruit Fly Research and Development in Africa—Towards a Sustainable Management Strategy to Improve Horticulture.

[4] Ekesi S., De Meyer M., Mohamed S.A., Virgilio M., Borgemeister C. (2016). Taxonomy, ecology, and management of native and exotic fruit fly species in Africa. Annu. Rev. Entomol..

[5] Baker R., Gilioli G., Behring C., Candiani D., Gogin A., Kaluski T., Kinkar M., Mosbach-Schulz O., Neri F.M., Preti S. (2019). Bactrocera dorsalis Pest Report to Support Ranking of EU Candidate Priority Pests.

[6] Zhao Z.H., Hui C., Plant R.E., Su M., Papadopoulos N.T., Carpenter T.E., Li Z.H., Carey J.R. (2019). The failure of success: Cyclic recurrences of a globally invasive pest. Ecol. Appl..

[7] Vitiello M., Benedetta F.d., Gargiulo S., Griffo R., Nugnes F., Bernardo U. (2020). *Bactrocera dorsalis* in Campania: Insediamento o incurione?. Entomata.

[8] De Villiers M., Hattingh V., Kriticos D.J., Brunel S., Vayssières J.F., Sinzogan A., Billah M., Mohamed S.A., Mwatawala M., Abdelgader H. (2016). The potential distribution of *Bactrocera dorsalis*: Considering phenology and irrigation patterns. Bull. Entomol. Res..

[9] Qin Y., Wang C., Zhao Z., Pan X., Li Z. (2019). Climate change impacts on the global potential geographical distribution of the agricultural invasive pest, *Bactrocera dorsalis* (Hendel) (Diptera: Tephritidae). Climatic Chang..

[10] Dominiak B.C., Mapson R. (2017). Revised distribution of *Bactrocera tryoni* in eastern Australia and effect on possible incursions of Mediterranean fruit fly: Development of Australia’s eastern trading block. J. Econ. Entomol..

[11] Liu H., Zhang D.-J., Xu Y.-J., Wang L., Cheng D.-F., Qi Y.-X., Zeng L., Lu Y. (2019). Invasion, expansion, and control of *Bactrocera dorsalis* (Hendel) in China. J. Integr.Agric..

[12] Ni W.L., Li Z.H., Chen H.J., Wan F.H., Qu W.W., Zhang Z., Kriticos D.J. (2012). Including climate change in pest risk assessment: The peach fruit fly, *Bactrocera zonata* (Diptera: Tephritidae). Bull. Entomol. Res..

[13] Zingore K.M., Sithole G., Abdel-Rahman E.M., Mohamed S.A., Ekesi S., Tanga C.M., Mahmoud M.E.E. (2020). Global risk of invasion by *Bactrocera zonata*: Implications on horticultural crop production under changing climatic conditions. PLoS ONE.

[14] Castrignanὸ A., Boccaccio L., Cohen Y., Nestel D., Kounatidis I., Papadopoulos N.T., De Benedetto D., Mavragani-Tsipidou P. (2012). Spatio-temporal population dynamics and area-wide delineation of *Bactrocera oleae* monitoring zones using multi-variate geostatistics. Precision Agric..

[15] Kean J.M., Stringer L.D. (2019). Optimising the seasonal deployment of surveillance traps for detection of incipient pest invasions. Crop Prot..

[16] Garcia Adeva J.J., Botha J.H., Reynolds M. (2012). A simulation modelling approach to forecast establishment and spread of *Bactrocera* fruit flies. Ecol. Model..

[17] van Klinken R., Murray J.V., Garcia J.N., Clarke A.R. (2019). Scale-appropriate spatial modelling to support area-wide management of a polyphagous fruit fly (Diptera: Tephritidae). Ann. Appl. Biol..

[18] Grechi I., Preterre A.-L., Caillat A., Chiroleu F., Ratnadass A. (2021). Linking mango infestation by fruit flies to fruit maturity and fly pressure: A prerequisite to improve fruit fly damage management via harvest timing optimization. Crop Prot..

[19] Vargas R.I., Piñero J.C., Leblanc L. (2015). An overview of pest species of *Bactrocera* fruit flies (Diptera: Tephritidae) and the integration of biopesticides with other biological approaches for their management with a focus on the Pacific region. Insects.

[20] Vargas R.I., Piñero J.C., Leblanc L., Manoukis N.C., Mau R.F.L., Ekesi S., Mohamed S.A., Meyer M.D. (2016). Area-wide management of fruit flies (Diptera: Tephritidae) in Hawaii. Fruit Fly Research and Development in Africa—Towards a Sustainable Management Strategy to Improve Horticulture.

[21] Meats A. (1981). The bioclimatic potential of the Queensland fruit fly, *Dacus tryoni*, in Australia. Proc. Ecol. Soc. Aust..

[22] Yonow T., Zalucki M.P., Sutherst R.W., Dominiak B.C., Maywald G.F., Maelzer D.A., Kriticos D.J. (2004). Modelling the population dynamics of the Queensland fruit fly, *Bactrocera* (*Dacus*) *tryoni*: A cohort-based approach incorporating the effects of weather. Ecol. Model..

[23] Peng C., Hui Y., Jianhong L. (2006). Population dynamics of *Bactrocera dorsalis* (Diptera: Tephritidae) and analysis of the factors influencing the population in Ruili, Yunnan Province, China. Acta Ecol. Sinica.

[24] Fiaboe K.K.M., Kekeunou S., Nanga S.N., Kuate A.F., Tonnang H.E.Z., Gnanvossou D., Hanna R. (2021). Temperature-based phenology model to predict the development, survival, and reproduction of the oriental fruit fly *Bactrocera dorsalis*. J. Therm. Biol..

[25] Ibrahim E.A., Salifu D., Mwalili S., Dubois T., Collins R., Tonnang H.E.Z. (2022). An expert system for insect pest population dynamics prediction. Comp. Electron. Agric..

[26] Jiang J.A., Syue C.H., Wang C.H., Liao M.S., Shieh J.S., Wang J.C. (2022). Precisely forecasting population dynamics of agricultural pests based on an interval type-2 fuzzy logic system: Case study for oriental fruit flies and the tobacco cutworms. Precision Agric..

[27] Susanto A., Permana A.D., Subahar T.S., Soesilohadi R.C.H., Leksono A.S., Fernandes A.A.R. (2022). Population dynamics and projections of fruit flies *Bactrocera dorsalis* and *B. carambolae* in Indonesian mango plantation. Agric. Nat. Res..

[28] Wolda H. (1983). “Long-term” stability of tropical insect populations. Res. Pop. Ecol..

[29] Wolda H. (1988). Insect Seasonality: Why?. Annu. Rev. Ecol. Syst..

[30] Braby M. (1995). Seasonal-changes in relative abundance and spatial-distribution of Australian lowland tropical Satyrine butterflies. Aust. J. Zool..

[31] Murphy P.G., Lugo A.E. (1986). Ecology of tropical dry forest. Annu. Rev. Ecol. Syst..

[32] Boulter S.L., Kitching R.L., Howlett B.G. (2006). Family, visitors and the weather: Patterns of flowering in tropical rain forests of northern *Aust*. J. Ecol..

[33] Wolda H. (1978). Seasonal fluctuations in rainfall, food and abundance of tropical insects. J. Anim. Ecol..

[34] Frith C.B., Frith D.W. (1985). Seasonality of insect abundance in an Australian upland tropical rainforest. Aust. J. Ecol..

[35] Williams S.E., Middleton J. (2007). Climatic seasonality, resource bottlenecks, and abundance of rainforest birds: Implications for global climate change. Divers. Distrib..

[36] Hodek I., Hodková M. (1988). Multiple role of temperature during insect diapause: A review. Entomol. Exp. Appl..

[37] Koštál V. (2006). Eco-physiological phases of insect diapause. J. Insect Physiol..

[38] Hahn D.A., Denlinger D.L. (2011). Energetics of insect diapause. Annu. Rev. Entomol..

[39] Wolda H., Denlinger D.L. (1984). Diapause in a large aggregation of a tropical beetle. Ecol. Entomol..

[40] Denlinger D.L. (1986). Dormancy in tropical insects. Annu. Rev. Entomol..

[41] Jones R.E. (1987). Reproductive strategies for the seasonal tropics. Internat. J. Trop. Insect Sci..

[42] Singtripop T., Wanichacheewa S., Tsuzuki S., Sakurai S. (1999). Larval growth and diapause in a tropical moth, *Omphisa fuscidentalis* Hampson. Zool. Sci..

[43] Claret J., Carton Y. (1980). Diapause in a tropical species, *Cothonaspis boulardi* (Parasitic Hymenoptera). Oecologia.

[44] Denlinger D.L. (1979). Pupal diapause in tropical flesh flies: Environmental and endocrine regulation, metabolic rate and genetic selection. Biol. Bull..

[45] Zonato V., Collins L., Pegoraro M., Tauber E., Kyriacou C.P. (2017). Is diapause an ancient adaptation in *Drosophila*?. J. Insect Physiol..

[46] Halali S., Brakefield P.M., Collins S.C., Brattström O. (2019). To mate, or not to mate: The evolution of reproductive diapause facilitates insect radiation into African savannahs in the Late Miocene. J. Anim. Ecol..

[47] Drew R.A.I. (1989). The tropical fruit flies (Diptera: Tephritidae: Dacinae) of the Australasian and Oceanian regions. Mem. Qld. Mus..

[48] Sultana S., Baumgartner J.B., Dominiak B.C., Royer J.E., Beaumont L.J. (2017). Potential impacts of climate change on habitat suitability for the Queensland fruit fly. Sci. Rep..

[49] Clarke A.R., Merkel K., Hulthen A.D., Schwarzmueller F. (2019). *Bactrocera tryoni* (Froggatt) (Diptera: Tephritidae) overwintering: An overview. Austral Entomol..

[50] Fletcher B.S. (1975). Temperature-regulated changes in the ovaries of overwintering females of the Queensland fruit fly, *Dacus tryoni*. Aust. J. Zool..

[51] Fletcher B.S. (1979). The overwintering survival of adults of the Queensland fruit fly, *Dacus tryoni*, under natural conditions. Aust. J. Zool..

[52] Fletcher B.S. (1986). The overwintering strategy of the Queensland fruit fly, *Dacus tryoni*. Fruit Flies: Proceedings of the Second International Symposium.

[53] Meats A. (1973). The abolition by low ambient temperature of tarsal inhibition of flight in certain Diptera. Search.

[54] Meats A. (1973). Rapid acclimatization to low temperature in the Queensland fruit fly, *Dacus tryoni*. J. Insect Physiol..

[55] Meats A. (1976). Developmental and long term acclimation to cold by the Queensland fruit fly (*Dacus tryoni*) at constant and fluctuating temperatures. J. Insect Physiol..

[56] Meats A. (1976). Seasonal trends in acclimatization to cold in the Queensland fruit fly (*Dacus tryoni*, Diptera) and their prediction by means of a physiological model fed with climatological data. Oecologia.

[57] Meats A. (1976). Thresholds for cold-torpor and cold-survival in the Queensland fruit fly, and predictability of rates of change in survival threshold. J. Insect Physiol..

[58] Meats A., Khoo K.C. (1976). The dynamics of ovarian maturation and oocyte resorption in the Queensland fruit fly, *Dacus tryoni*, in daily rhythmic and constant temperature regimes. Physiol. Entomol..

[59] Meats A., Robinson A.S., Hooper G.H.S. (1989). Acclimation, activity levels and survival. Fruit Flies: Biology, Natural Enemies and Control.

[60] Meats A., Fitt G.P. (1987). Survival of repeated frosts by the Queensland fruit fly, *Dacus tryoni*: Experiments in laboratory simulated climates with either step or ramp fluctuations in temperature. Entomol. Exp. Appl..

[61] Muthuthantri S., Maelzer D., Zalucki M.P., Clarke A.R. (2010). The seasonal phenology of *Bactrocera tryoni* (Froggatt) (Diptera: Tephritidae) in Queensland. Aust. J. Entomol..

[62] Lloyd A.C., Hamacek E.L., Kopittke R.A., Peek T., Wyatt P.M., Neale C.J., Eelkema M., Gu H.N. (2010). Area-wide management of fruit flies (Diptera: Tephritidae) in the Central Burnett district of Queensland, Australia. Crop Prot..

[63] Subramaniam S., Jackson K., Lloyd A., Rosemary K., Hamacek E., Kreymborg D. (2011). Alternative Fruit Fly Control and Market Access for Capsicums and Tomatoes: A System Approach for Tomato and Capsicum Production in Bowen.

[64] Drew R.A.I., Zalucki M.P., Hooper G. (1984). Ecological studies of eastern Australian fruit flies (Diptera: Tephritidae) in their endemic habitat. I. Temporal variation in abundance. Oecologia.

[65] Yonow T., Sutherst R.W. (1998). The geographical distribution of the Queensland fruit fly, *Bactrocera* (*Dacus*) *tryoni*, in relation to climate. Aust. J. Agric. Res..

[66] Streatfield C. (2009). Demography and population genetic structure of Uromys caudimaculatus. Ph.D. Thesis.

[67] Gallego D., García-Herrera R., Peña-Ortiz C., Ribera P. (2017). The steady enhancement of the Australian Summer Monsoon in the last 200 years. Sci. Rep..

[68] Cooper W., Cooper W.T. (2013). Australian Rainforest Fruits: A Field Guide.

[69] Innis G.J. (1989). Feeding ecology of fruit pigeons in subtropical rainforests of South-Eastern Queensland. Aust. Wildlife Res..

[70] Drew R.A.I., Courtice A.C., Teakle D.S. (1983). Bacteria as a natural source of food for adult fruit flies (Diptera: Tephritidae). Oecologia.

[71] Deutscher A.T., Reynolds O.L., Chapman T.A. (2017). Yeast: An overlooked component of *Bactrocera tryoni* (Diptera: Tephritidae) larval gut microbiota. J. Econ. Entomol..

[72] Akami M., Andongma A.A., Zhengzhong C., Nan J., Khaeso K., Jurkevitch E., Niu C.-Y., Yuval B. (2019). Intestinal bacteria modulate the foraging behavior of the oriental fruit fly *Bactrocera dorsalis* (Diptera: Tephritidae). PLoS ONE.

[73] de Jager E.S., Wehner F.C., Korsten L. (2001). Microbial ecology of the mango phylloplane. Micro. Ecol..

[74] Jumpponen A., Jones K.L. (2010). Seasonally dynamic fungal communities in the *Quercus macrocarpa* phyllosphere differ between urban and nonurban environments. New Phytol..

[75] Izuno A., Tanabe A.S., Toju H., Yamasaki M., Indrioko S., Isagi Y. (2016). Structure of phyllosphere fungal communities in a tropical dipterocarp plantation: A massively parallel next-generation sequencing analysis. Mycoscience.

[76] O’Loughlin G.T., East R.A., Meats A. (1984). Survival, development rates and generation times of the Queensland fruit fly, *Dacus tryoni*, in a marginally favourable climate: Experiments in Victoria. Aust. J. Zool..

[77] Tasnin S.M., Bode M., Merkel K., Clarke A.R. (2021). A polyphagous, tropical insect herbivore shows strong seasonality in age-structure and longevity despite temperature and hosts not being limiting. Sci. Rep..

[78] Fanson B.G., Sundaralingam S., Jiang L., Dominiak B.C., D’Arcy G. (2014). A review of 16 years of quality control parameters at a mass-rearing facility producing Queensland fruit fly, *Bactrocera tryoni*. Entomol. Exp. Appl..

[79] Dominiak B.C., Sundaralingam S., Jiang L., Nicol H.I. (2014). Longevity of mass-produced *Bactrocera tryoni* (Diptera: Tephritidae) held without food or water. J. Econ. Entomol..

[80] Dominiak B.C., Gillespie P.S., Loecker H., Reid N., Sharma N. (2021). Seasonal weight fluctuations in wild Queensland fruit fly *Bactrocera tryoni* (Froggatt) (Diptera: Tephritidae) may be a survival mechanism. Crop Prot..

[81] Pritchard G. (1970). The ecology of a natural population of Queensland fruit fly, *Dacus tryoni* III. The maturation of female flies in relation to temperature. Aust. J. Zool..

[82] Bateman M.A., Sonleitner F.J. (1967). The ecology of a natural population of the Queensland fruit fly, *Dacus tryoni* I. The parameters of the pupal and adult populations during a single season. Aust. J. Zool..

[83] Merkel K., Schwarzmueller F., Hulthen A.D., Schellhorn N., Williams D., Clarke A.R. (2019). Temperature effects on “overwintering” phenology of a polyphagous, tropical fruit fly (Tephritidae) at the subtropical/temperate interface. J. Appl. Entomol..

[84] Tasnin S.M., Kay B.J., Peek T., Merkel K., Clarke A.R. (2021). Age-related changes in reproductive potential of Queensland fruit fly. J. Insect Physiol..

[85] Jarvis H. (1922). Fruit fly investigations [First progress report]. Qld Agric. J..

[86] Jarvis H. (1922). Fruit fly investigations [Second progress report]. Qld Agric. J..

[87] Flatt T., Amdam G.V., Kirkwood T.B., Omholt S.W. (2013). Life-history evolution and the polyphenic regulation of somatic maintenance and survival. Quart. Rev. Biol..

[88] Pener M.P. (1992). Environmental cues, endocrine factors, and reproductive diapause in male insects. Chronobiol. Intern..

[89] Tatar M., Yin C.M. (2001). Slow aging during insect reproductive diapause: Why butterflies, grasshoppers and flies are like worms. Exper. Gerontol..

[90] Wallingford A.K., Loeb G.M. (2016). Developmental acclimation of *Drosophila suzukii* (Diptera: Drosophilidae) and its effect on diapause and winter stress tolerance. Environ. Entomol..

[91] Hodek I. (2002). Controversial aspects of diapause development. Europ. J. Entomol..

[92] Rossi-Stacconi M.V., Kaur R., Mazzoni V., Ometto L., Grassi A., Gottardello A., Rota-Stabelli O., Anfora G. (2016). Multiple lines of evidence for reproductive winter diapause in the invasive pest *Drosophila suzukii*: Useful clues for control strategies. J. Pest Sci..

[93] Pieloor M.J., Seymour J.E. (2001). Factors affecting adult diapause initiation in the tropical butterfly *Hypolimnas bolina* L.(Lepidoptera: Nymphalidae). Aust. J. Entomol..

[94] Yap S., Fanson B.G., Taylor P.W. (2015). Mating reverses actuarial aging in female Queensland fruit flies. PLoS ONE.

[95] Nylin S. (2013). Induction of diapause and seasonal morphs in butterflies and other insects: Knowns, unknowns and the challenge of integration. Physiol. Entomol..

[96] Fitt G.P. (1981). The ecology of Northern Australian Dacinae (Diptera: Tephritidae) I. Host phenology and utilization of *Opilia amentacea* Roxb. (Opiliaceae) by *Dacus* (*Bactrocera*) *opiliae* Drew & Hardy, with notes on some other species. Aust. J. Zool..

[97] Fitt G.P. (1983). The influence of seasonal climatic factors on the development of the methyl eugenol response in male *Dacus opiliae*. Entomol. Exp. Et Appl..

[98] Fletcher B.S. (1987). The biology of *Dacinae* fruit flies. Annu. Rev. Entomol..

[99] Syed R.A. (1968). Studies on the Ecology of Some Important Species of Fruit Flies and Their Natural Enemies in West Pakistan.

[100] Hancock D.L. (1985). New species and records of African Dacinae (Diptera: Tephritidae). Arnoldia Zimbabwe.

[101] Kishimoto-Yamada K., Itioka T. (2015). How much have we learned about seasonality in tropical insect abundance since Wolda (1988)?. Entomol. Sci..

[102] Theron C., Manrakhan A., Weldon C.W. (2017). Host use of the oriental fruit fly, *Bactrocera dorsalis* (Hendel) (Diptera: Tephritidae), in South Africa. J. Appl. Entomol..

[103] Denlinger D.L. (1978). The developmental response of flesh flies (Diptera: Sarcophagidae) to tropical seasons. Oecologia.

[104] Madder M., Speybroech N., Brandt J., Tirry L., Hodek I., Berkvens D. (2002). Geographic variation in diapause response of adult *Rhipicephalus appendiculatus* ticks. Exp. Appl. Acarol..

[105] Denlinger D.L., Hahn D.A., Merlin C., Holzapfel C.M., Bradshaw W. (2017). Keeping time without a spine: What can the insect clock teach us about seasonal adaptation?. Phil. Trans. Royal Soc. (B).

[106] Abro Z.-U.-A., Baloch N., Memon R.M., Khuhro N.H. (2021). Population fluctuation of *Bactrocera zonata* and *Bactrocera dorsalis* in guava orchard agro-ecosystem in Sindh Region. Pakistan J. Zool..

[107] Boinahadji A.K., Coly E.V., Dieng E.O., Diome T., Sembene P.M. (2019). Interactions between the oriental fruit fly *Bactrocera dorsalis* (Diptera, Tephritidae) and its host plants range in the Niayes area in Senegal. J. Entomol. Zool. Studies.

[108] Chen P., Ye H. (2007). Population dynamics of *Bactrocera dorsalis* (Diptera: Tephritidae) and analysis of factors influencing populations in Baoshanba, Yunnan, China. Entomol. Sci..

[109] Danjuma S., Boonrotpong S., Thaochan N., Permkam S., Satasook C. (2014). Seasonality of the Asian papaya fruit fly *Bactrocera papayae* Drew and Hancock (Diptera: Tephritidae) on guava *Psidium guajava* in peninsular Thailand. J. Entomol. Zool. Studies.

[110] Gnanvossou D., Hanna R., Goergen G., Salifu D., Tanga C.M., Mohamed S.A., Ekesi S. (2017). Diversity and seasonal abundance of tephritid fruit flies in three agro-ecosystems in Benin, West Africa. J. Appl. Entomol..

[111] Orankanok W., Chinvinikjul S., Thanaphum S., Sitilob P., Enkerlin W.R., Vreysen M.J.B., Robinson A.S., Hendrichs J. (2007). Area-wide integrated control of Oriental fruit fly *Bactrocera dorsalis* and guava fruit fly *Bactrocera correcta* in Thailand. Area-Wide Control of Insect Pests: From Research to Field Implementation.

[112] Umeh V., Onukwu D., Ekesi S., Mohamed S.A., Meyer M.D. (2016). Integrated management of fruit flies—Case studies from Nigeria. Fruit Fly Research and Development in Africa—Towards a Sustainable Management Strategy to Improve Horticulture.

[113] Vayssieres J.F., De Meyer M., Ouagoussounon I., Sinzogan A., Adandonon A., Korie S., Wargui R., Anato F., Houngbo H., Didier C. (2015). Seasonal abundance of mango fruit flies (Diptera: Tephritidae) and ecological implications for their management in mango and cashew orchards in Benin (Centre & North). J. Econ. Entomol..

[114] Bess H.A., Haramoto F.H. (1961). Contributions to the Biology and Ecology of the Oriental fruit FLY, Dacus dorsalis Hendel (Diptera: Tephritidae), in Hawaii.

[115] Haramoto F.H., Bess H.A. (1970). Recent studies on the abundance of the Oriental and Mediterranean fruit flies and the status of their parasites. Proc. Hawaiin Entomol. Soc..

[116] Newell I.M., Haramoto F.H. (1968). Biotic factors influencing populations of *Dacus dorsalis* in Hawaii. Proc. Hawaiin Entomol. Soc..

[117] Samayoa A.C., Choi K.S., Wang Y.-S., Hwang S.-Y., Huang Y.-B., Ahn J.J. (2018). Thermal effects on the development of *Bactrocera dorsalis* (Hendel) (Diptera: Tephritidae) and model validation in Taiwan. Phytoparasitica.

[118] Choi K.S., Samayoa A.C., Hwang S.-Y., Huang Y.-B., Ahn J.J. (2020). Thermal effect on the fecundity and longevity of *Bactrocera dorsalis* adults and their improved oviposition model. PLoS ONE.

[119] Vargas R.I., Stark J.D., Nishida T. (1990). Population dynamics, habitat preference, and seasonal distribution patterns of Oriental fruit fly and Melon fly (Diptera: Tephritidae) in an agricultural area. Environ. Entomol..

[120] Kamala Jayanthi P.D., Verghese A. (2011). Host-plant phenology and weather based forecasting models for population prediction of the oriental fruit fly, *Bactrocera dorsalis* Hendel. Crop Prot..

[121] Kamala Jayanthi P.D., Verghese A., Sreekanth P.D. (2011). Predicting the oriental fruit fly *Bactrocera dorsalis* (Diptera: Tephritidae) trap catch using artificial neural networks: A case study. Inter. J. Trop. Insect Sci..

[122] Kamal Jayanthi K.P., Verghes A., Sreekanth P.D., Arthikirubha R., Jayasimha G.T. (2014). Temperature dependent phenological synchrony between host-fruit availability and occurrence of Oreintal fruit fly, *Bactrocera dorsalis*, a crucial link to study climate change. Indian J. Plant Prot..

[123] Chuang C.-L., Yang E.-C., Tseng C.-L., Chen C.-P., Lien G.-S., Jiang J.-A. (2014). Toward anticipating pest responses to fruit farms: Revealing factors influencing the population dynamics of the Oriental fruit fly via automatic field monitoring. Comp. Electron. Agric..

[124] Hong S.C., Magarey R.D., Borchert D.M., Vargas R.I., Souder S.K. (2015). Site-specific temporal and spatial validation of a generic plant pest forecast system with observations of *Bactrocera dorsalis* (Oriental fruit fly). Neobiota.

[125] Hussain D., Saleem M., Abbas M., Ali Q., Qasim M., Hafeez F., Ashrif M., Zubair M., Saleem M.J., Ghouse G. (2022). Monitoring and management of fruit fly population using the male annihilation technique with different types of cost-effective traps in guava orchards of Punjab, Pakistan. Inter. J.Pest Manag..

[126] Diouf E.G., Brévault T., Ndiaye S., Faye E., Chailleux A., Diatta P., Piou C. (2022). An agent-based model to simulate the boosted Sterile Insect Technique for fruit fly management. Biol. Model..

[127] Balagawi S., Jackson K., Haq I.U., Hood-Nowotny R., Resch C., Clarke A.R. (2014). Nutritional status and the foraging behaviour of *Bactrocera tryoni* with particular reference to protein bait spray. Physiol. Entomol..

[128] Biosecurity Queensland (2021). ICA-34. Pre-Harvest Field Control and Inspection of Strawberries Vs. 4.

[129] Gu H. (2010). Alternative fruit fly treatment for interstate market access for strawberries. Final report for project BS06002.

[130] Kean J.M. (2016). Modelling winter survival, mating and trapping of Queensland fruit fly in Auckland, New Zealand. New Zealand Plant Prot..

[131] Schwarzmueller F., Schellhorn N.A., Parry H. (2019). Resource landscapes and movement strategy shape Queensland Fruit Fly population dynamics. Landsc. Ecol..

[132] Dong Z., He Y., Ren Y., Wang G., Chu D. (2022). Seasonal and year-round distributions of *Bactrocera dorsalis* (Hendel) and its risk to temperate fruits under climate change. Insects.

[133] Skendžić S., Zovko M., Živković I.P., Lešić V., Lemić D. (2021). Effect of climate change on introduced and native agricultural invasive insect pests in Europe. Insects.

[134] Gutierrez A.P., Ponti L., Neteler M., Suckling D.M., Cure J.R. (2021). Invasive potential of tropical fruit flies in temperate regions under climate change. Commun. Biol..

[135] Meats A. (1983). Critical periods for developmental acclimation in the Queensland fruit fly, *Dacus tryoni*. J. Insect Physiol..

[136] Denlinger D.L., Lee R.E., Denlinger D.L. (1991). Relationship between cold hardiness and diapause. Insects at Low Temperature.

[137] Stephens A.E.A., Kriticos D.J., Leriche A. (2007). The current and future potential geographical distribution of the oriental fruit fly, *Bactrocera dorsalis* (Diptera: Tephritidae). Bull. Entomol. Res..

[138] Hernandez-Ortiz V., Perez-Alonso R. (1993). The natural host plants of *Anastrepha* (Diptera: Tephritidae) in a tropical rain forest of Mexico. Florida Entomol..

[139] Aluja M., Celedonio-Hurtado H., Liedo P., Cabrera M., Castillo F., Guillén J., Rios E. (1996). Seasonal population fluctuations and ecological implications for management of *Anastrepha* fruit flies (Diptera: Tephritidae) in commercial mango orchards in Southern Mexico. J. Econ. Entomol..

[140] Ovruski S.M., Norrbom A.L., Schliserman P., Aluja M. (2005). Biology and taxonomy of *Rhagoletotrypeta* (Diptera: Tephritidae): A new species from Cuba and new host plant, parasitoid, and distribution records from Northwestern Argentina. Ann. Entomol. Soc. Am..

[141] Aluja M., Lopez M., Sivinski J. (1998). Ecological evidence for diapause in four native and one exotic species of larval-pupal fruit fly (Diptera: Tephritidae) parasitoids in tropical environments. Ann. Entomol. Soc. Am..

[142] Rungrojwanich K., Walter G.H. (2000). The Australian fruit fly parasitoid *Diachasmimorpha kraussii* (Fullaway): Life history, ovipositional patterns, distribution and hosts (Hymenoptera: Braconidae: Opiinae). Pan-Pac. Entomol..

[143] Ovruski S.M., Schliserman P., Aluja M. (2016). Occurrence of diapause in neotropical parasitoids attacking *Anastrepha fraterculus* (Diptera: Tephritidae) in a subtropical rainforest from Argentina. Austral Entomol..

[144] Danks H.V. (1987). Insect Dormancy: An Ecological Perspective.

[145] Godfray H.C.J. (1994). Parasitoids: Behavioral and Evolutionary Ecology.

